# Diosmetin Affects Gene Expression on Human Lung Adenocarcinoma Cells

**DOI:** 10.1155/2022/5482148

**Published:** 2022-05-20

**Authors:** Changshan Song, Shunfu Deng, Hui Hu, Zheng Zheng, Bairu Shen, Xuhui Wu, Minqian Huang, Jiaqing Wang, Zhenyu Wang

**Affiliations:** ^1^Department of Thoracic Surgery, Foshan Clinical Medical School of Guangzhou University of Chinese Medicine, Guangdong 528000, China; ^2^Department of Thoracic Surgery, Affiliated Foshan Foxing Chancheng Hospital of Guangdong Medical University, Guangdong 528000, China; ^3^Department of Thoracic Surgery, Foshan Foxing Chancheng Hospital, Guangdong 528000, China

## Abstract

**Objective:**

This study was aimed at investigating the effects of diosmetin (a natural flavonoid) on the gene expression of human lung adenocarcinoma (LUAD) cells.

**Methods:**

HCC827 and A549 cells were used. MTT and colony formation assay were used to investigate the effects of diosmetin on cell proliferation and colony forming activity. The expression of mRNA, microRNA, and lncRNA in HCC827 and A549 cell lines after diosmetin treatment was measured using DNA microarray, microRNA chromatin immunoprecipitation assay (ChIP), and long noncoding RNA (lncRNA) ChIP. Part of the results were cross-validated by quantitative reverse transcription PCR (RT-qPCR), while some others were analyzed using bioinformatic tools.

**Results:**

Diosmetin inhibited proliferation and colony formation of HCC827 and A549 cells. Investigation on gene expression profiles of A549 and HCC827 cells revealed that compared with the control group, diosmetin can up- or downregulated the expression of mRNAs, microRNAs, and lncRNAs. The top three candidates in each RNA category were cross-validated by RT-qPCR, from which single peaks were observed in the melt curves, showing a great specificity. After a comprehensive selection of the results from the mRNA ChIP, we performed GO and KEGG functional clustering analyses on the differentially expressed genes.

**Conclusion:**

Diosmetin treatment induced gene expression of A549 and HCC827 cells. Our results will provide guidance for development of new diagnostic and therapeutic targets.

## 1. Introduction

Lung cancer is one of the most common types of malignant tumors. It is the third most common cancer after breast cancer and prostate cancer, which seriously threatens human health and quality of life. Lung cancer has been the focus of attention of cancer researchers around the world [1]. Due to the lack of specific clinical manifestations, early-stage lung cancer can be easily confused with general respiratory tract inflammatory diseases, which leads to low rate of diagnosis. Most of the lung cancer cases are found in the middle and late stages, which has lost the best opportunity for surgery. For patients with surgical indications, surgery includes tumor resection and lymph node dissection. Surgery is currently the preferred treatment for lung cancer. However, according to statistics, after the surgical treatment, the 5-year survival rate of lung cancer is only 15%-20%. In the past two decades, anticancer drugs have been developed. Natural ingredients in Chinese herbal medicine, including some flavonoids and semisynthetic taxane derivatives, play a very important role in improving antitumor efficacy and developing specific chemotherapeutic drugs. Current chemotherapeutic drugs for lung adenocarcinoma (LUAD) have no selectivity, low efficacy, severe toxicity, and side effects. Therefore, it is very important to find a safe and effective chemotherapeutic drug with more targets and less side effects.

Diosmetin is a monomeric flavonoid polyphenol compound. It is derived from various plants such as *Galium verum*, chrysanthemum, lemon, and citrus. In recent years, increasing numbers of studies on diosmetin have shown its effects on anti-inflammation, antitumor, antithrombosis, immune regulation, free radical scavenging, and other pharmacological effects [2, 3]. Ma and Zhang [4] found that diosmetin inhibits the proliferation of liver cancer cells by targeting checkpoint kinase 2 (Chk2) and promotes cancer cell apoptosis and cell cycle arrest. Yan et al. [5] found that diosmetin may activate E-cadherin expression and inhibit transforming growth factor-*β* (TGF-*β*) signaling pathway, thereby inhibiting the growth of glioma cells. Diosmetin is cytotoxic to a variety of tumor cells, and the mechanism of action of diosmetin in different cancer cells might not be the same. This might be related to the difference in the intracellular glycolipidation cite of diosmetin.

Researchers found that the expression of genes, such as epidermal growth factor receptor (EGFR), Kirsten rat sarcoma viral oncogene (*KRAS)*, and mitogen-activated protein kinase (MAPK) signaling pathways, is different in LUAD tissue and normal lung tissue [6–8]. Analyzing the RNA ChIP for gene expression in LUAD, it was found that some genes were related to poor prognosis. Earlier study using RNA ChIP found that the expression of TTF-1 gene was different in LUAD and small-cell lung cancer (SCLC) [9]. In addition to differences in gene expression, the role of noncoding RNA in tumors has also received increasing attention. Some researchers have observed that the expression of microRNAs in PC-9 and HCC-827 gefitinib-resistant cells is different, and miR-1-3p and miR-206 can significantly increase the sensitivity of PC-9 and HCC-827 cells to gefitinib [10]. Some researchers have used ChIP to observe the changes in the expression of microRNAs in LUAD cells under hypoxia. In recent years, accumulating evidence has shown that lncRNAs play an important role in tumor development. In previous studies, we have observed that diosmetin has an inhibitory effect on LUAD HCC827 and A549 cells both in vitro and in vivo. In this research, we use RNA ChIP to evaluate the regulatory effect of diosmetin on LUAD HCC827 and A549 cells to understand the mechanism of action of diosmetin in repressing LUAD.

## 2. Materials and Methods

### 2.1. Cell Culture

The LUAD cell line HCC827 and A549 were provided by the Department of Thoracic Surgery, Foshan Clinical Medical College, Guangzhou University of Chinese Medicine. The cells were cultured in RPMI-1640 complete medium containing 10% fetal bovine serum, penicillin, and streptomycin and placed in a 5% CO2, saturated humidity, and 37°C constant temperature incubator. The cells in the logarithmic phase were taken for the experiment.

### 2.2. MTT Assay

HCC827 and A549 cells (2 × 103 per well) were seeded into 96-well plates and cultured in complete culture medium. After 12-hour incubation, the cells were incubated in the presence or absence of different concentrations of diosmetin for 24 hours. Then, 25 mL of MTT (5 mg/mL) was added and incubated for an additional 3 hours. After that, the medium was replaced with 100 mL DMSO to dissolve the crystals by shaking the plate for 10 minutes. Absorbance at 450 nm was read with a microplate reader (ELx800, BioTek Instruments Inc., USA).

### 2.3. Colony Formation Assay

HCC827 and A549 cells were seeded into 6-well plates (500/well) and placed in a 5% CO_2_, saturated humidity, and 37°C constant temperature incubator. After 12 hours, the cells were incubated in the presence or absence of different concentrations of diosmetin for 14 days. Then, the cells were fixed by 4% paraformaldehyde for 10 min and stained by 0.1% crystal violet for 5 min. The number of colonies (at least 50 cells) was counted.

### 2.4. RNA Extraction and Quality Control and Microarray

HCC827 and A549 cells were seeded into 6-well plates (1 × 10^5^/well). After 12 hours, the cells were incubated in the presence or absence of different concentrations of diosmetin for 24 hours. Then, total RNA was extracted from cells using TRIzol reagent according to the manufacturer's instructions. NanoDrop was used to detect the purity and concentration of the extracted RNA. The microarray experiments were performed using the Affymetrix GeneChip Human Transcriptome Array (Affymetrix, MA, USA). The microarray comprises more than 40,000 noncoding transcripts (including lncRNAs, miRNAs, and small nucleolar RNAs). The cDNA labeling, microarray analysis, and bioinformatics analysis were performed by Genminix Informatics (Genminix, Shanghai, China). The aberrantly expressed transcripts (lncRNAs, miRNAs, and mRNAs) were identified using *P* values <0.05.

### 2.5. Purification of Total RNA

RNA was purified by using the Ambion miRVana miRNA Isolation Kit. Briefly, the RNA was dissolved in 50 *μ*l RNase-free water, and 200 *μ*l lysis/binding buffer and 24 *μ*l miRNA homogenate additive were added and mixed. The mixture was centrifuged and solution collected. It was put on ice bath for 10 min, added 330 *μ*l of ethanol, mixed and transferred to the RNA purification column, centrifuged, and the effluent discarded. The purification column was washed with 700 *μ*l miRNA Wash Solution 1, then 50 *μ*l of preheated miRNA Elution Solution was added to the purification column, let it stand for 1 min, and the effluent was discarded after centrifugation.

### 2.6. RT-qPCR Reaction

The RT-qPCR reaction mixture (Table 1) was added to the AB TaqMan Human microRNA array plate and centrifuged at room temperature and 1200 rpm for 1 min, repeat for one time. The AB TaqMan Human MicroRNA Array plate was sealed.

### 2.7. ChIP Result Clustered Analysis

The results of miRNA expression were normalized. Cluster 3.0 was used to determine the cluster, and cluster analysis chart was drawn. The diosmetin group was compared with the control group with a 2-fold difference after normalization as the screening criterion for expression difference.

### 2.8. RT-qPCR Verified Target Gene Expression in Cell Lines

The RT-qPCR reaction condition was 95°C for 5 min, 95°C for 15 s, 60°C for 15 s, and 72°C for 32 s, 40 cycles. After the reaction, the results were analyzed by the comparative Ct value method, and the formula is as follows: △△Ct = [CtmiR − 223 − CtU6]Diosmetin/control − [Cttarget − CtU6]NP69. The expression level of target gene was calculated using the 2-△△Ct method in the diosmetin-treated cells relative to the control cells.

### 2.9. Statistical Analysis

ChIP data was analyzed via Cluster 3.0 and Significance Analysis of Microarrays (SAM, version 2.1). The difference of 2 times in expression was used as the criterion for identifying differentially expressed genes.

## 3. Result

### 3.1. Diosmetin Inhibited the Viability and Colony Formation of HCC827 and A549 Cells

**Figure 1:**
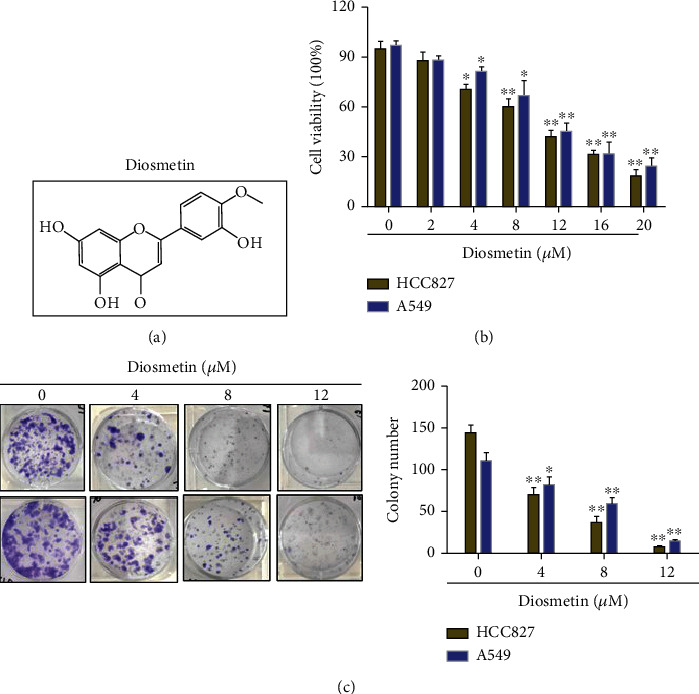
Diosmetin inhibited viability and colony formation of human lung adenocarcinoma HCC827 and A549 cells. (a) The structure of diosmetin. (b) The effect of diosmetin on the viability of HCC827 and A549 cells by MTT assay. (c) Representative images of colonies of HCC827 and A549 cells from six-well plates where cells were treated with 0, 2, 4, and 8 *μ*M of diosmetin for 14 d. ^∗^*P* < 0.05 and ^∗∗^*P* < 0.01.

Previous studies have showed that diosmetin (Figure 1(a)), as a natural flavonoid present in legumes, olive leaves, and citrus plants, has anticancer activity. In the present study, the cell viability was assessed using MTT assays. The results showed that diosmetin treatment for 24 hours significantly inhibited cell growth in a dose-dependent manner (Figure 1(b)). Furthermore, colony formation assay showed that the proliferation of both HCC827 and A549 cells was inhibited by diosmetin with increasing concentration. Quantification analysis of the colony numbers showed that diosmetin inhibited cell proliferation in a dose-dependent manner (Figure 1(c)). These results indicated that diosmetin has the inhibitory effects on the proliferation and cell viability of lung adenocarcinoma cells.

### 3.2. ChIP Analysis

**Figure 2:**
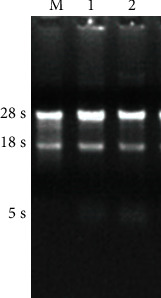
Agarose gel electrophoresis to detect the integrity of total RNA in 3 kinds of cells. M: HeLa cell control; 1: A549 cells; 2: HCC827 cells.

In order to investigate the effects of diosmetin on the gene expression of HCC827 and A549 cells, total RNA was isolated from cells, and the results showed that the RNA concentration was in the range of 100 ng/*μ*l~ 500 ng/*μ*l, and the values at A260/A280 were between 1.8 and 2.0. RNA samples were subjected to formaldehyde denaturation agarose gel electrophoresis. 5s rRNA, 18s rRNA, and 28s rRNA bands are complete and clear. 28s:18s rRNA band brightness is greater than or close to 2 : 1, indicating that the RNA was not degraded (Figure 2).

**Figure 3:**
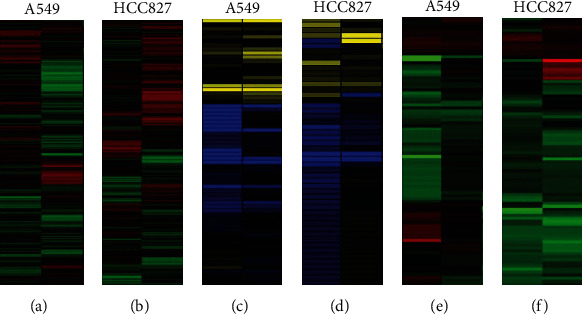
After the A549 and HCC827 cells were treated with 4 *μ*M diosmetin, the expression of cellular mRNAs, microRNAs, and lncRNAs was analyzed by cluster analysis. (a) A549 cell mRNAs, (b) HCC827 cell mRNAs, (c) A549 cell miRNAs, (d) HCC827 cell miRNAs, (e) A549 cell lncRNAs, and (f) HCC827 cell lncRNAs.

The microarray results of mRNAs, microRNAs, and lncRNAs after applying diosmetin on A549 and HCC827 cells were clustered with cluster3.0, and part of the cluster analysis results is shown in Figure 3.

### 3.3. Diosmetin Affected the Expression of mRNAs, miRNAs, and lncRNAs in A549 and HCC827 Cells

The expression of mRNAs, miRNAs, and lncRNAs was different in A549 and HCC827 cells treated with diosmetin. Compared with the control group, the top 10 up- and downregulated mRNAs, microRNAs, and lncRNAs in A549 and HCC827 cells are shown in Tables 2–7.

### 3.4. RT-qPCR Verification

**Figure 4:**
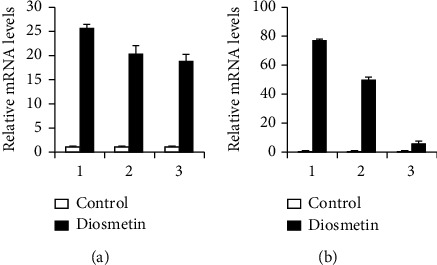
RT-qPCR verification on the mRNAs of A549 and HCC827 cells treated with 4 *μ*M diosmetin. (a) A549 cells: 1: glucoside xylosyltransferase 2; 2: interleukin 7 receptor; 3: collagen, type V, alpha. (b) HCC827 cells: 1: deiodinase, iodothyronine, type II 2; 2: zinc finger protein 804A; 3: p53-responsive gene 1.

**Figure 5:**
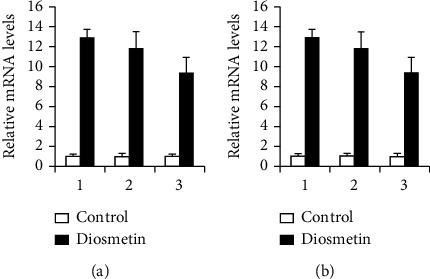
RT-qPCR verification on the miRNAs of A549 and HCC827 cells treated with 4 *μ*M diosmetin. (a) A549 cells: 1: miR-144; 2: miR-6858; 3: miR-6867. (b) HCC827 cells: 1: miR-144 2; 2: miR-6867; 3: miR-4669.

**Figure 6:**
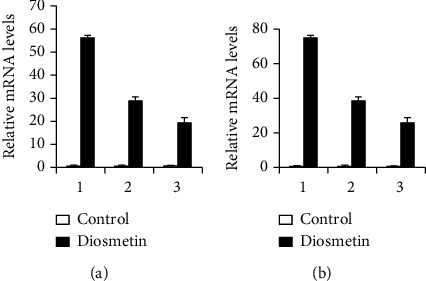
RT-qPCR verification on the lncRNAs of A549 and HCC827 cells treated with 4 *μ*M diosmetin. (a) A549 cells: 1: RNA50955; 2: RNA41013; 3: RNA54473. (b) HCC827 cells: 1: RNA51025 2; 2: RNA50931; 3: RNA62890.

Three mRNAs, miRNAs, and lncRNAs with the highest expression in each group were selected, and the ChIP results were verified by the RT-qPCR method. The RT-qPCR results are consistent with the ChIP (Figures 4–6).

### 3.5. Clustered Analysis of ChIP Results

**Figure 7:**
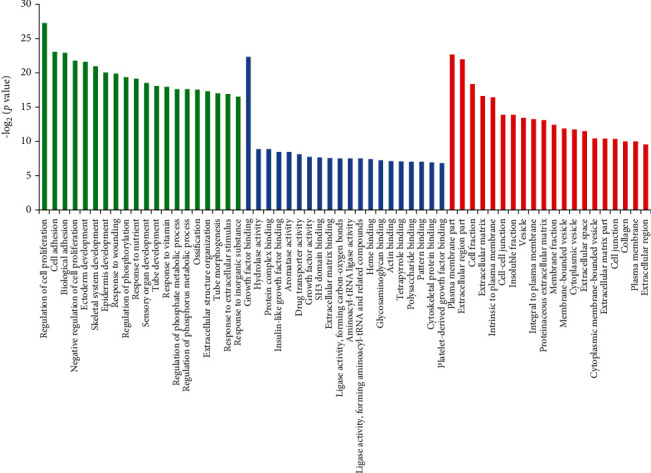
GO function cluster analysis of mRNAs expression ChIP after A549 and HCC827cells were treated with 4 *μ*M diosmetin. Green is biological process, blue is cell location, and red is molecular function.

**Figure 8:**
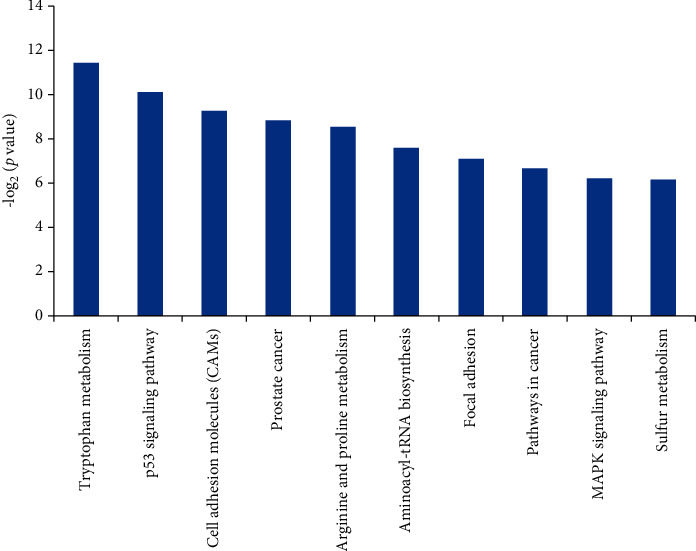
The GO function clustering analysis on A549 and HCC827cells treated with 4 *μ*M diosmetin.

After the A549 and HCC827 cells were treated with 4 *μ*M diosmetin, the expression of mRNAs ChIP results was screened and integrated. The results obtained were subjected to GO and KEGG functional cluster analyses. It can be seen from Figures 7 and 8 that some differentially expressed gene clusters were enriched, which provides inspiration for further studies.

## 4. Discussion

LUAD is one of the most common cancers threatening human health. The main clinical treatment for LUAD is surgery removal combined with radiotherapy and/or chemotherapy. Due to the low sensitivity and side effect of radiotherapy and/or chemotherapy, the current clinical approaches are often not satisfactory. Thus, development of new antitumor drugs for LUAD has attracted a lot of attention. Diosmetin has an antitumor effect that closely related to the activity of cytochrome P450. Diosmetin is an inhibitor of the cytochrome P450 1A1 and 1B1 by acting as an antagonist of aryl hydrocarbon receptor and affects the activity of cytochrome P450 1A1. Thus, diosmetin can mediate the drug metabolism by inhibiting cytochrome P450 2C9 and 2C8 [11]. The antitumor effects of diosmetin through cytochrome P450 family 1 (CYP1) could be seen in three aspects: (1) inhibiting CYP1 enzyme activity; (2) acts as the substrate of CYP1; and (3) the combined effect of 1 and 2. A recent study revealed that in breast cancer, diosmetin can inhibit cell proliferation by inducing cell cycle arrest. Additionally, diosmetin has the antiproliferation and proapoptosis effects in MDA-MB-231 cells [12]. In ovarian cancer, it was found that ovarian cancer apoptosis is induced by activating reactive oxygen species and inhibiting NRF2 gene [13]. In lung cancer, some studies indicate that diosmetin can selectively induce cell apoptosis through ROS accumulation by disrupting the PI3K/Akt/GSK-3*β*/Nrf2 pathway and enhance the efficacy of paclitaxel in NSCLC cells [14]. However, a comprehensive understanding of diosmetin effect in LUAD is still lacking.

Noncoded RNA (ncRNA) is a class of noncoding transcripts involved in regulation of a variety of biological processes such as cell growth, proliferation, apoptosis, and differentiation. It affects target gene after reverse transcription, thereby regulating the translational level and expression of that gene. In 180,000 transcripts of human cells, approximately 20,000 are protein coding, and the remaining 160,000 were noncoded transcripts. miRNA and lncRNA have the strongest association with lung cancer. miRNA is a set of noncoded RNAs between 20 NT and 25 NT that inhibits mRNA translation or enhances mRNA regulatory associated gene expression, thereby participating in cell proliferation, differentiation, and apoptosis [15]. Studies have shown that miR-33a-5p can activate Wnt/*β*-catenin signals through the JPX/Twist1 axis to participate in the EMT process, making lung cancer cells easy to metastasize [16, 17]. lncRNA is a class of noncoded RNA larger than 200 nT. It can be used as sponges of miRNA, or combined with enhancers to help their activity. For instance, it promotes the formation of chromatin loops and the recruitment of remodeling complexes. Zhou et al. demonstrated that lncRNA DLEU2 is upregulated in NSCLC tissues and cells, and by targeting miR-30c-5p, it can enhance the proliferation, invasion, and migration and reduce apoptosis of A549 and LLC cells [18]. L. Xu et al. revealed that compared to A549 cells, lncRNA SNHG14 and HOXB13 were upregulated, while miR-133a was downregulated in A549/DDP cells [19]. Knockdown of SNHG14 or overexpression of miR-133a has been shown to increase the DDP sensitivity of A549/DDP cells. Thus, this study uses RNA ChIP to observe the cell epigenetics changes after A549 and HCC827 cells were treated with diosmetin. Furthermore, bioinformatics was used to explore gene expression pathway regulated by diosmetin. In this study, the differentially expressed mRNAs, microRNAs, and lncRNAs caused by the effects of diosmetin on A549 and HCC827 cells were screened using RNA ChIP. The results were verified by RT-qPCR and both results are consistent. These results lay the foundation for future clinical uses of diosmetin.

In addition to diosmetin, other natural or synthetic compounds also have inhibitory effects on LUAD. In terms of inducing apoptosis, for example, curcumin and dimethylolcoumarin can partially inhibit the ERK/MAPK signaling pathway through the ROS-independent mitochondrial pathway to induce cell apoptosis. Digitalis flavonoids induce mitochondrial apoptosis in LUAD cells. Myricetol extracted from bayberry bark can inhibit the proliferation and induce apoptosis of A549 cells. Leucocephalus can regulate c-Myc/S phase protein, WD repeat sequence protein, 7/histidine deacetylase, and other signaling pathway to induce apoptosis of LUAD cells. Pine cones induce apoptosis of LUAD cells by activating caspase-3. In A549 cells, the epidermal growth factor inhibitor gefitinib can upregulate apoptotic molecules and downregulate antiapoptotic molecules through a p53-dependent pathway. Other molecules, oxidized carotene, can inhibit proliferation of A549 cells. Green tea can upregulate the expression of annexin 1 to promote actin rearrangement in A549 cells. The proanthocyanidins in grape seeds can inhibit the metastasis of non-small-cell lung cancer by inhibiting the activities of nitric oxide, guanylate cyclase, and ERK1/2. Schizandrin inhibits the proliferation of A549 cells by controlling cell cycle and inducing cell apoptosis.

Taken together, diosmetin affects the gene expression and proliferation of human lung adenocarcinoma A549 and HCC827 cells. This study provides a rationale for using diosmetin for lung cancer treatment.

## Figures and Tables

**Table 1:** RT-qPCR reaction system.

Reagent	Standard volume
TaqMan universal PCR master mix II, no UNG (2×)	450 *μ*l
Megaplex RT product	6 *μ*l
Nuclease-free water	444 *μ*l
Volume of mix	900 *μ*l

**Table 2:** Top 10 differentially expressed mRNAs in A549 cells treated with diosmetin.

Upregulated mRNAs	Times of difference	Downregulated mRNAs	Times of difference
Glucoside xylosyltransferase 2	25.52	Phospholipase A2, group VI	6.90
Interleukin 7 receptor	20.37	Mucin 6, oligomeric mucus/gel forming	6.86
Collagen, type V, alpha 1	18.84	3-hydroxy-3-methylglutaryl-CoA reductase	6.84
Bone morphogenetic protein 5	13.83	Cytochrome P450, family 51, subfamily A, polypeptide 1	6.19
Carboxypeptidase A4	12.71	Squalene epoxidase	5.55
Fibroblast growth factor 21	12.50	Low density lipoprotein receptor	4.24
Insulin-like growth factor 1 (somatomedin C)	9.64	G protein-coupled receptor 180	4.04
Interleukin 6 (interferon, beta 2)	6.52	Neuropilin 1	3.88
p53-responsive gene 1	5.86	Myelin basic protein	3.85
GTP binding protein 2	2.80	Glutamate receptor interacting protein 2	3.75

**Table 3:** Top 10 differentially expressed mRNAs in HCC827 cells treated with diosmetin.

Upregulated mRNAs	Times of difference	Downregulated mRNAs	Times of difference
Deiodinase, iodothyronine, type II	77.62	Calbindin 2	39.31
Zinc finger protein 804A	50.17	Keratin 5	16.11
p53-responsive gene 1	5.86	Insulin induced gene 1	11.14
CD22 molecule	4.39	Calpain 8	10.00
Low density lipoprotein receptor-related protein 1	4.34	Amphiregulin	8.29
Quiescin Q6 sulfhydryl oxidase 1	3.30	Keratin 12	3.73
ATPase, class II, type 9A	3.25	Neuropilin (NRP) and tolloid- (TLL-) like 2	3.69
CD274 molecule	3.09	GATA zinc finger domain containing 2A	3.67
SMAD family member 9	3.03	Cyclin E2	3.62
Tumor necrosis factor receptor superfamily, member 13C	2.78	Pyruvate dehydrogenase phosphatase catalytic subunit 2	3.57

**Table 4:** Top 10 differentially expressed miRNAs in A549 cells treated with diosmetin.

Upregulated miRNAs	Times of difference	Downregulated miRNAs	Times of difference
miR-144-3p	12.93	miR-6075	18.74
miR-6858-3p	11.83	miR-4707-5p	10.12
miR-6867-5p	9.42	miR-7641	6.70
miR-1234-3p	8.35	miR-654-5p	6.66
miR-4669	8.23	miR-6790-5p	5.00
miR-188-5p	6.17	miR-1973	3.98
miR-4499	4.72	miR-7855-5p	3.85
miR-4433a-5p	4.69	Let-7c-5p	3.67
miR-483-5p	4.51	miR-4286	3.40
miR-4769-3p	3.83	miR-4713-3p	3.36

**Table 5:** Top 10 differentially expressed miRNAs in HCC827 cells treated with diosmetin.

Upregulated miRNAs	Times of difference	Downregulated miRNAs	Times of difference
miR-144-3p	12.93	miR-3188	15.06
miR-6867-5p	9.42	miR-1260a	8.43
miR-4669	8.23	miR-15b-5p	6.89
miR-4499	4.72	Let-7a-5p	5.56
miR-483-5p	4.51	miR-20b-5p	4.09
miR-1273e	3.64	miR-4485-3p	3.96
miR-6740-5p	3.57	miR-328-3p	3.67
miR-7110-5p	1.64	miR-6073	3.55
miR-6812-5p	1.61	miR-6892-5p	3.38
miR-6124	1.56	miR-6807-5p	3.29

**Table 6:** Top 10 differentially expressed lncRNAs in A549 cells treated with diosmetin.

Upregulated lncRNAs	Times of difference	Downregulated lncRNAs	Times of difference
RNA50955	74.15	RNA162924	194.48
RNA41013	32.84	RNA47224	80.16
RNA54473	20.43	RNA47232	31.50
RNA176433	15.00	RNA47233	28.72
RNA47987	13.30	RNA47236	27.98
RNA164874	10.50	RNA35973	25.39
RNA39763	9.73	RNA175965	23.08
RNA52786	8.65	RNA34218	19.20
RNA163263	8.07	RNA36405	16.25
RNA50955	4.15	RNA41014	14.98

**Table 7:** Top 10 differentially expressed lncRNAs in HCC827 cells treated with diosmetin.

Upregulated lncRNAs	Times of difference	Downregulated lncRNAs	Times of difference
RNA51025	56.49	RNA63048	164.38
RNA50931	29.10	RNA47235	31.66
RNA62890	20.11	RNA44429	28.81
RNA164103	15.33	RNA61561	28.62
RNA47521	13.76	RNA47234	27.73
RNA59320	12.89	RNA177059	23.16
RNA46553	9.88	RNA160049	22.05
RNA96388	8.43	RNA54714	18.21
RNA47856	8.03	RNA40894	15.39
RNA42876	8.02	RNA177063	14.93

## Data Availability

Emails could be sent to the address songcs2000@163.com to obtain the shared data.
